# Elevation of Cleaved p18 Bax Levels Associated with the Kinetics of Neuronal Cell Death during Japanese Encephalitis Virus Infection

**DOI:** 10.3390/ijms20205016

**Published:** 2019-10-10

**Authors:** Prapimpun Wongchitrat, Arisara Samutpong, Hatairat Lerdsamran, Jarunee Prasertsopon, Montri Yasawong, Piyarat Govitrapong, Pilaipan Puthavathana, Kuntida Kitidee

**Affiliations:** 1 Center for Research and Innovation, Faculty of Medical Technology, Mahidol University, Salaya, Nakhon Pathom 73170, Thailand; 2 Chulabhorn Graduate Institute, Chulabhorn Royal Academy, Bangkok 10210, Thailand; 3 Research Center for Neuroscience, Institute of Molecular Biosciences, Mahidol University, Salaya, Nakhon Pathom 73170, Thailand

**Keywords:** Japanese encephalitis virus, kinetic viral replication, neuronal cell death, apoptosis, Bcl-2, Bax

## Abstract

Japanese encephalitis virus (JEV) infection induces uncontrolled neuronal apoptosis, leading to irreversible brain damage. However, the mechanism of JEV-induced neuronal apoptosis has not been clearly elucidated. This study aimed to investigate both virus replication and neuronal cell apoptosis during JEV infection in human neuroblastoma SH-SY5Y cells. As a result, the kinetic productions of new viral progeny were time- and dose-dependent. The stimulation of SH-SY5Y cell apoptosis was dependent on the multiplicity of infections (MOIs) and infection periods, particularly during the late period of infection. Interestingly, we observed that of full-length Bax (p21 Bax) level started to decrease, which corresponded to the increased level of its cleaved form (p18 Bax). The formation of p18 Bax resulting in cytochrome *c* release into the cytosol appeared to correlate with JEV-induced apoptotic cell death together with the activation of caspase-3/7 activity, especially during the late stage of a robust viral infection. Therefore, our results suggest another possible mechanism of JEV-induced apoptotic cell death via the induction of the proteolysis of endogenous p21 Bax to generate p18 Bax. This finding could be a new avenue to facilitate novel drug discovery for the further development of therapeutic treatments that could relieve neuronal damage from JEV infection.

## 1. Introduction

Japanese encephalitis virus (JEV), a neurotropic mosquito-borne flavivirus, is known as the cause of acute viral encephalitis. Japanese encephalitis has a high mortality rate in humans, and almost 60% of the global population is at risk of exposure to JEV, especially in Asia [1]. Approximately 25% of Japanese encephalitis patients die, while 30–50% of survivors experience serious neurologic, cognitive, or psychiatric complications [2]. A permanent neuronal abnormality subsequently affects the quality of a patient’s life. Despite the importance of Japanese encephalitis, little is known about the pathogenesis underlying JEV infection.

Japanese encephalitis virus is a positive-sense single-stranded RNA virus, and its genome encodes three structural proteins (C, prM, and E) and seven non-structural proteins (NS1, NS2A, NS2B, NS3, NS4A, NS4B, NS5) [3]. The main JEV transmission cycle involves *Culex tritaeniorhynchus* mosquitoes and similar species that lay eggs in rice paddies and other open water resources, with pigs and aquatic birds as the principal vertebrate amplifying hosts. Humans are generally considered dead-end JEV hosts [4].

Studies from other flaviviruses have revealed a possible mechanism of JEV entering the central nervous system (CNS). After a mosquito bite, JEV may replicate in the cells of the dermal tissue before reaching lymphoid organs, and then the virus enters into the blood circulation and crosses the blood–brain barrier (BBB) to the CNS [2]. This virus can infect several neural cells, including neurons, astrocytes, microglia, and vascular endothelial cells, where the presence of JEV antigens has been detected [5,6]. The invasion of the CNS by JEV is associated with neurodegeneration by generating oxidative stress of infected neuron cells and triggering a robust inflammatory response that leads to brain neuronal cell death [7,8].

Japanese encephalitis virus infection causes neuronal apoptosis, which is an important process attributed to JEV pathogenesis in the CNS. Previous studies have demonstrated the elevation of oxidants such as ROS and NO radicals after JEV infection [9]. A decline in intracellular antioxidants was observed during JEV infection [10]. Several JEV infection models exhibit the activation of apoptosis signaling molecules, including the induction of B cell lymphoma-2 (Bcl-2) family proteins, which are regulators of apoptosis [11,12,13]. This group of proteins comprises anti-apoptotic molecules, such as Bcl-2, and proapoptotic members, such as Bax. These two molecules interact with each other and play a crucial role in controlling cell life and death [14]. Apoptosis induction by viral infection is caused by the increase in Bax translocation from the cytosol to mitochondria to promote the release of cytochrome *c* (Cyt *c*) and induce caspase cascade activation [15,16,17,18]. In addition to the proapoptotic effect of Bax translocation, the proteolytic cleavage of Bax at the N-terminus by calpain generates a potent proapoptotic 18 kDa fragment (p18 Bax) that appears to enhance apoptotic cell death [19,20,21]. Moreover, previous reports demonstrated that the NS3 and NS2B-NS3 protease and helicase domains could induce cell apoptosis via the caspase-9/-3 cascade pathway [11,22]. However, the mechanisms by which virus–host interactions induce apoptosis pathways during JEV infection are unclear.

In the present study, we investigated the kinetics of apoptosis and the expression of the apoptosis-related proteins Bcl-2, Bax, and caspase-3 during JEV infection in human neuroblastoma SH-SY5Y cells. Our results showed the dynamics of JEV replication in human neuroblastoma cells. During JEV infection, the JEV-infected cells were induced to undergo apoptosis depending on different virus doses and infection periods. Japanese encephalitis virus appeared to trigger cell apoptosis during the late period of infection. The increase in p18 Bax seemed to correlate with the induction of apoptosis during JEV infection, especially at a high multiplicity of infection (MOI). Our findings suggest that the induction of neuronal cell death by JEV is associated with the mitochondria-mediated apoptosis pathway and may be involved in the formation of an N-terminal truncated version of Bax.

## 2. Results

### 2.1. JEV Infection of SH-SY5Y Cells

**Figure 1:**
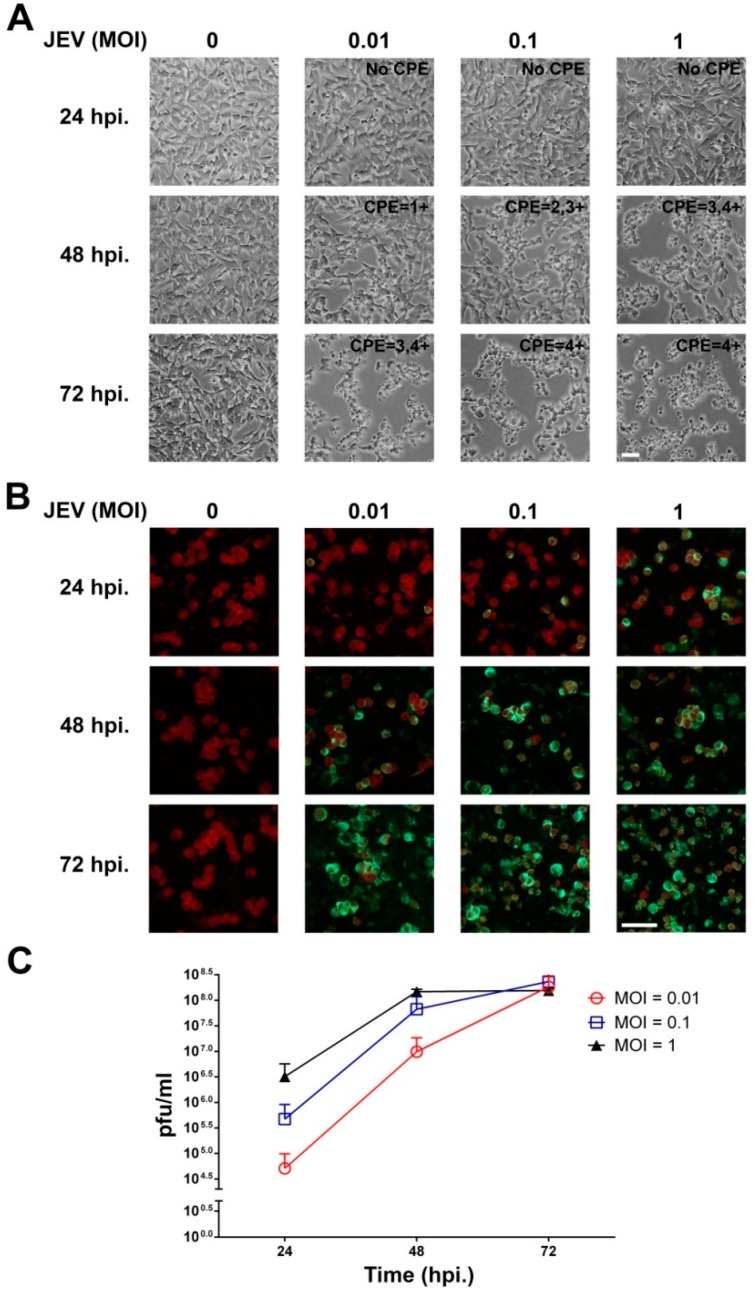
Japanese encephalitis virus (JEV) infection in SH-SY5Y human neuroblastoma cells. SH-SY5Y cells were inoculated with JEV at a multiplicity of infection (MOI) of 0.01, 0.1, and 1 for 24, 48, and 72 h post infection (hpi). (**A**) Cytopathic effect (CPE) in JEV-infected SH-SY5Y cells with different MOIs (magnification, 20×). (**B**) Immunofluorescence analysis of viral envelope protein (green) expression in JEV-infected SH-SY5Y cells. The cell location is shown by Evan blue counterstaining (red) (magnification, 40×). Scale bar are 10 µm. Images are representative of three independent experiments. (**C**) Replication kinetics of JEV in SH-SY5Y cells. Culture supernatants were collected at 24, 48, and 72 hpi, and virus titers were determined by a plaque assay using Vero cells. The results shown are the mean ± SD of three independent experiments.

In this study, to ensure that JEV was able to infect and replicate in human neuroblastoma cells, SH-SY5Y cells were inoculated with different MOIs of JEV and incubated for 24, 48, and 72 h. As shown in Figure 1A, JEV infection of SH-SY5Y cells at an MOI of 0.01, 0.1, and 1 induced cytopathic effect (CPE), including rounding up and aggregation at 48 hpi, and the CPE was markedly observed at 72 hpi when compared with the uninfected cells. In addition, the green fluorescent JEV-infected cells were first observed at 24 hpi for all MOIs, and the number of infected cells gradually increased at 72 hpi (Figure 1B). Uninfected cells showed no fluorescence. Thus, the SH-SY5Y cells were susceptible to JEV infection. To study the ability of JEV to replicate in neuronal cells, the production of new viral progeny was determined by plaque assay. As shown in Figure 1C, JEV replicated in a dose-dependent manner starting at 24 hpi. The virus titers of both MOIs of 0.01 and 0.1 were continuously increased up to the maximum titer of approximately 10^8^ pfu/mL at 72 hpi. At an MOI of 1, JEV replicated efficiently and reached the highest titer of approximately 10^8^ pfu/mL by 48 hpi. At 72 hpi of 1 MOI, the virus titer seemed to plateau. This result demonstrated the replication kinetics of JEV in neuronal cells. Taken together, the human neuroblastoma SH-SH5Y cells were susceptible to JEV infection and allowed the virus to replicate because these cells were permissive cells.

### 2.2. JEV Infection Induces Cell Death in SH-SY5Y Cells

**Figure 2:**
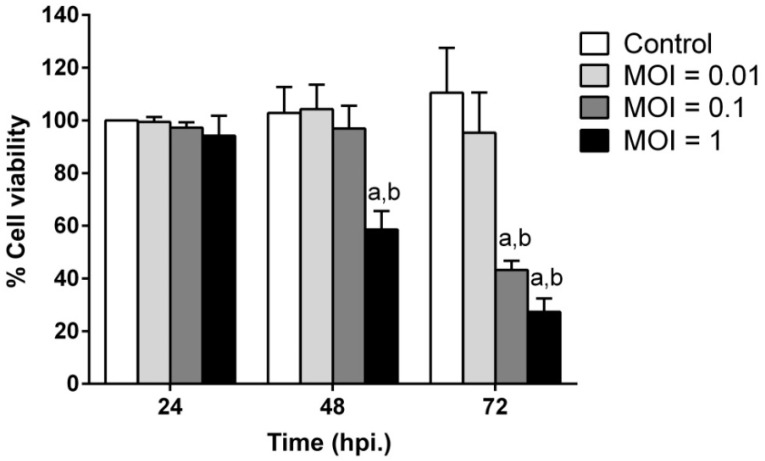
The effect of JEV infection on cell viability in SH-SY5Y human neuroblastoma cells. SH-SY5Y cells were infected with JEV at different MOIs, and the cell viability of infected cells was determined at the indicated time by a cell viability assay. The results shown are the mean ± SD of three independent experiments. Two-way ANOVA and Tukey–Kramer multiple comparisons tests were performed for statistical analysis. ^a^ *p* < 0.01, compared to the control at each time point. ^b^ *p* < 0.01, compared with the same MOI at 24 hpi.

In addition to the CPE caused by JEV infection, the induction of neuronal cell death by JEV infection in a time- and dose-dependent pattern was also determined by a cell viability assay (Figure 2). At 24 hpi, the cell viability of JEV-infected cells at all MOIs showed no significant difference in cell death when compared to uninfected cells. The percentage of cell death was significantly increased at 48 hpi for 1 MOI (*p* < 0.01) and 72 hpi for 0.1 MOI (*p* < 0.01) when compared to uninfected cells at each time point. The percentage of cell viability dramatically declined to less than 40% at 72 hpi for both MOIs of 0.1 and 1. No significant difference in cell viability was observed at any time point for a JEV MOI of 0.01 compared to uninfected cells.

### 2.3. JEV Infection Induces Apoptosis in SH-SY5Y Cells

**Figure 3:**
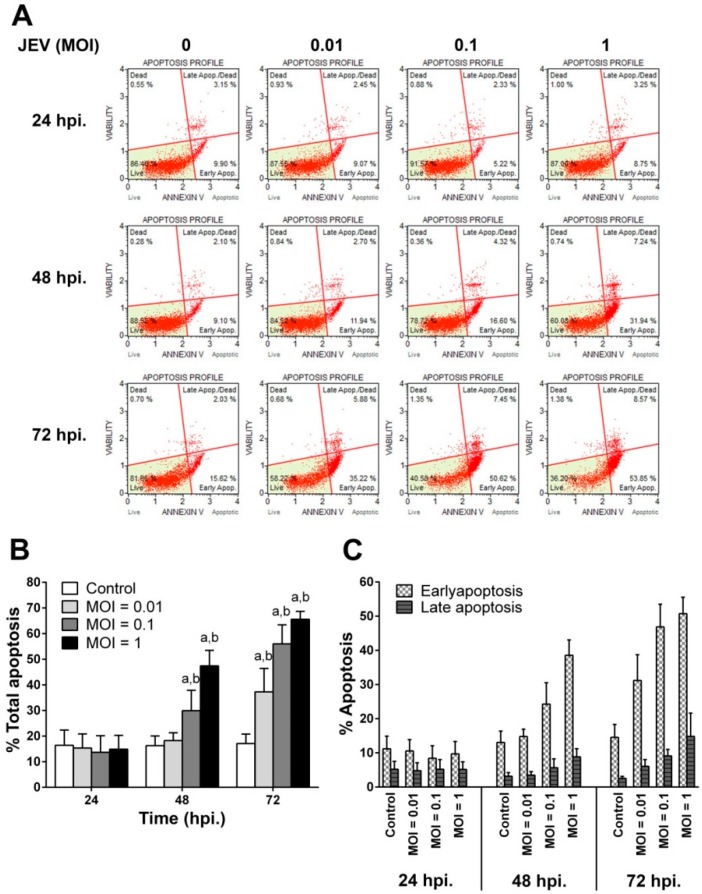
Induction of SH-SY5Y cell apoptosis by JEV infection. SH-SY5Y cells were infected with JEV at different MOIs, and the number of apoptotic cells were determined at the indicated times of 24, 48, and 72 hpi using the Muse^®^ Annexin-V and Dead Cell Kit. (**A**) Scatter plot of JEV-infected SH-SY5Y cells. The cells were divided into four different populations based on apoptosis/cell death staining. Quadrant 1 represents live cells, quadrant 2 represents early apoptotic cells, quadrant 3 represents late apoptotic cells, and quadrant 4 represents death cells. (**B**) The total apoptotic rate of SH-SY5Y cells during JEV infection. (**C**) The percentage of early and late apoptotic cells. Data are expressed as the mean ± SD of five independent experiments. Two-way ANOVA and Tukey–Kramer multiple comparisons tests were performed for statistical analysis. ^a^ *p* < 0.05, compared to the control at each time point. ^b^ *p* < 0.01, compared with the same MOI at 24 hpi.

To confirm that JEV-induced SH-SY5Y cell death was due to the fact of apoptosis, annexin V and 7-AAD staining of apoptotic cells was performed and analyzed by flow cytometry to differentiate the number of apoptotic cells and cell death (Figure 3). The scatter plot of JEV-infected SH-SY5Y cells at each time point after infection is shown in Figure 3A. At 24 hpi, the apoptosis of JEV-infected cells for all MOIs was equal to the apoptosis found in uninfected control cells. However, the rate of apoptosis significantly increased in both JEV 0.1 MOI (*p* < 0.05) and 1 MOI at 48 hpi (*p* < 0.05) when compared with the rate in the uninfected control cells (Figure 3B). After 72 hpi of JEV infection, the apoptosis rate markedly increased and reached a maximum level of 55.98 ± 3.33% at an MOI of 0.1 and 65.58 ± 1.39% at an MOI of 1 (Figure 3B). In addition, the percentage of annexin V-positive cells alone was higher than those of annexin V and 7-AAD-positive cells in all MOIs and periods of infection. This indicated that JEV could induce cells to undergo the early apoptosis stage rather than the late apoptosis stage (Figure 3C). The results suggested that the rate of neuronal cell apoptosis induced by JEV infection depended on the number of virus inoculations and the infection period. Notably, the pattern of cell apoptosis measured by flow cytometry correlated with that of cell death measured by cell viability assay for all MOIs during JEV infection.

### 2.4. Expression of Bcl-2 Family Members in JEV-Infected SH-SY5Y Cells

**Figure 4:**
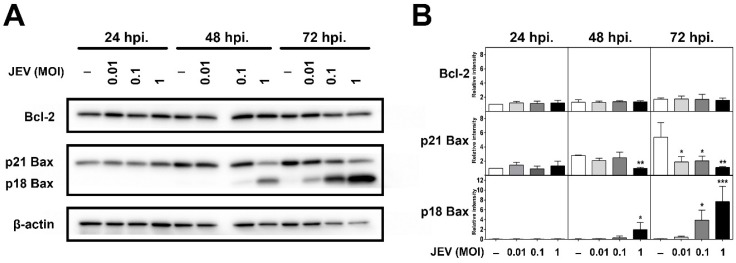
Protein expression profiles of Bcl-2 family members in JEV-infected SH-SY5Y cells. SH-SY5Y cells were infected with JEV at different MOIs, and total protein was extracted at the indicated times of 24, 48, and 72 hpi. (**A**) The immunoreactive detection and (**B**) respective quantification of Bcl-2, p21 Bax, and p18 Bax were analyzed by Western blot. The immunoblots shown are from one representative experiment. Densitometric analysis values represent mean ± SD from three independent experiments. One-way ANOVA and Tukey–Kramer multiple comparisons tests were performed for statistical analysis. * *p* < 0.05, ** *p* < 0.01, and *** *p* < 0.001, compared to the control at each time point.

Japanese encephalitis virus appeared to trigger apoptosis in SH-SY5Y cells during the late period of infection (after 24 h). To investigate whether JEV-induced cell death is related to the expression of Bcl-2 family members, we determined Bcl-2 and Bax expression levels in JEV-infected cells by Western blot analysis. As shown in Figure 4, the uninfected control cells revealed the endogenous expression levels of Bcl-2 and the full-length form of Bax (p21) at all time points. Anti-apoptotic protein Bcl-2 inhibits apoptosis by counteracting Bax [14]. The effect of JEV infection on Bcl-2 and Bax expression in SH-SY5Y cells is demonstrated as the immunoreactive bands (Figure 4A) and the respective quantification (Figure 4B). Our results revealed that the expression level of Bcl-2 was not affected by all MOIs during JEV infection at all time points. The pro-apoptosis protein Bax regulates the release of Cyt *c* from mitochondria and initiates apoptosis [23]. Uninfected cells showed an increase in full-length p21 Bax expression in a time-dependent manner. Japanese encephalitis virus-infected cells exhibited alterations in Bax expression. We observed that p21 Bax levels were not different for any MOI at 24 h after inoculation. The level of p21 Bax started to decrease from 48 to 72 hpi in cells infected with JEV MOI of 1, which corresponded to the increase in the levels of its cleaved form. In addition, we were able to detect the significant increasing levels of the cleaved form of Bax (p18) at the time when a high percentage of cell death occurred during JEV infection in SH-SY5Y cells. The p18 Bax fragment levels were upregulated by JEV infection, especially at the later stage of cell death, which was detected at 48 to 72 hpi following JEV infection. The levels of p18 Bax were significantly increased at a JEV MOI of 0.1 and 1 but not at an MOI of 0.01 compared to undetectable levels in the uninfected control cells (Figure 4B). The proteolytic cleavage of Bax occurred in a time- and dose-dependent pattern. The presence of Bax cleavage suggests that JEV-induced SH-SY5Y cell death is a mitochondria-mediated intrinsic apoptosis pathway.

### 2.5. JEV Infection Increases p18 Bax Accumulation in Mitochondria and Induced Cyt c Release into the Cytosol

**Figure 5:**
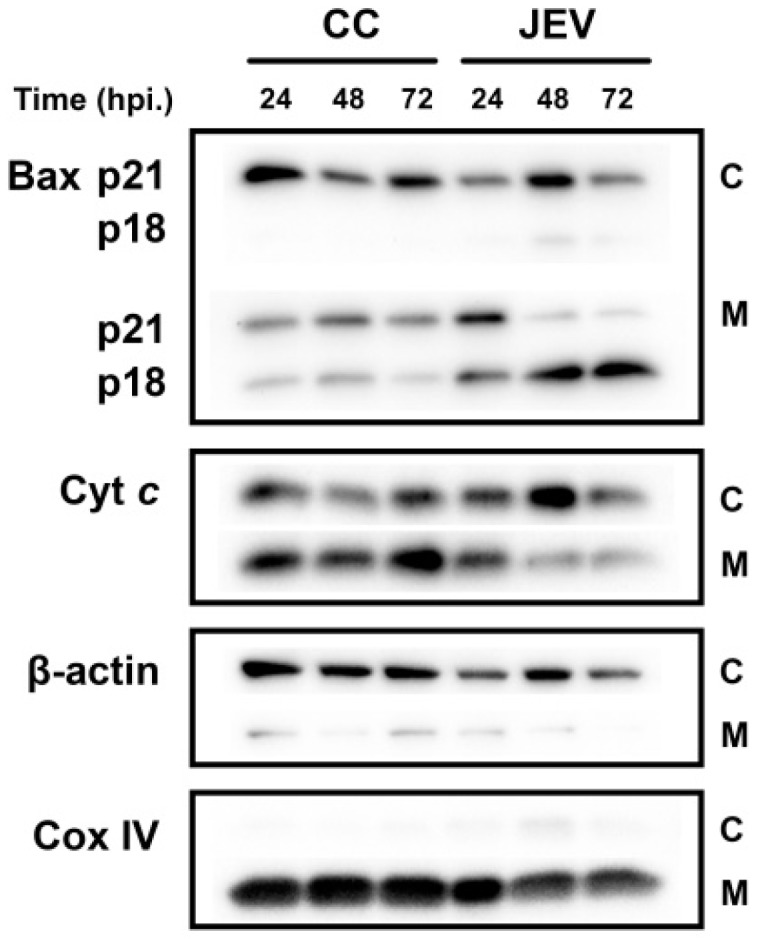
The effect of JEV infection on Bax and Cyt *c* translocation in SH-SY5Y cells. SH-SY5Y cells were infected with JEV at MOI of 1, and mitochondrial and cytosolic protein were extracted by subcellular fractionation at the indicated times of 24, 48, and 72 hpi. The protein translocation of Bax and Cyt *c* were analyzed by Western blot. C indicates cytoplasmic fraction and M indicates mitochondrial fraction. β-actin and Cox IV were used as a loading control and a purity of cytosolic and mitochondrial fraction, respectively.

According to the induction of the cleaved p18 Bax after JEV infection at high MOI, we then further investigated the localization of p18 Bax in cellular compartments of JEV-infected SH-SY5Y cells by subcellular fractionation and Western blotting (Figure 5). The results showed that the p18 Bax was exclusively detected in the mitochondria fraction of cells infected with JEV at an MOI of 1. The mitochondrial p18 Bax levels increased in a time-dependent manner while the full-length p21 Bax appeared to decrease in the mitochondria fraction over time. To further confirm that the increase of p18 Bax was responsible for the mitochondrial translocation of Cyt *c* into the cytosol, which is a hallmark of the mitochondria-dependent pathway of apoptosis, the release of Cyt *c* from the mitochondria was determined. As compared with the control group, the Cyt *c* levels in JEV-infected cells increased in the cytosol and decreased in the mitochondria also in a time-dependent manner. These results confirm that JEV infection induced apoptosis through the mitochondria-mediated intrinsic pathway by inducing the level of the cleaved p18 Bax which led to the release of Cyt *c* from mitochondria into cytoplasm.

### 2.6. JEV Infection Triggers Apoptosis by Inducing Caspase-3/7 Activity

**Figure 6:**
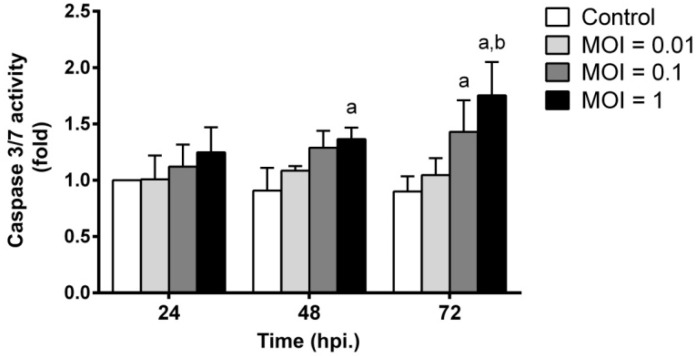
Induction of caspase-3/7 activity profile in SH-SY5Y cells by JEV infection. SH-SY5Y cells were infected with JEV at different MOIs, and the caspase activity of infected cells was determined at the indicated times of 24, 48, and 72 hpi by the Muse™ Caspase-3/7 Assay. Data are expressed as the mean ± SD of three independent experiments. Two-way ANOVA and Tukey–Kramer multiple comparisons tests were performed for statistical analysis. ^a^ *p* < 0.01, compared to the control at each time point. ^b^ *p* < 0.01, compared with the same MOI at 24 hpi.

Effector caspase-3 and -7 are downstream of death programs, and their activity plays important roles in the proteolytic cleavage of several cellular targets during apoptosis, leading to the activation of apoptotic cell death [24]. To evaluate potential apoptosis regulation triggered by JEV infection, caspase-3/7 activity was measured. The kinetics of caspase-3/7 activity induced by different MOIs of JEV are shown in Figure 6. Our results showed that there was no significant change in caspase-3/7 activity in JEV infection with a 0.01 MOI at any time point after infection compared to uninfected control cells. Japanese encephalitis virus infection at an MOI of 0.1 at 48 hpi initiated the induction of caspase-3/7 activity. The level of caspase-3/7 activity in JEV-infected cells at 72 hpi at a 0.1 MOI (*p* < 0.01) was significantly higher than that in uninfected control cells. In the case of infection with an MOI of 1, the activity of caspase-3/7 continually increased from 24 hpi and reached the highest activity at 72 hpi compared to the activity in uninfected cells at that time point (*p* < 0.01). Obviously, the activation of caspase-3/7 at 72 hpi was much higher during JEV infection with an MOI of 1 than that with an MOI of 0.01 (*p* < 0.01). These results suggest that JEV infection induced SH-SY5Y cell apoptosis mediated by caspase activation.

## 3. Discussion

Japanese encephalitis virus still circulates and causes viral encephalitis in many countries, particularly in Asia. Permanent neurological sequelae caused by JEV-induced neuronal cell death must be considered since this complication affects the quality of the patient’s life. In our study, we investigated the ability of JEV to infect, replicate, and induce cell apoptosis in human neuroblastoma SH-SY5Y cells. We found that SH-SY5Y cells were susceptible to the JEV strain Beijing-1. At low (0.01), medium (0.1), or high (1) MOI, CPE was first observed at 48 hpi (Figure 1A), although infected cells were detected at 24 hpi. Interestingly, at 72 hpi, the level of CPE rapidly changed in the JEV-infected cells at medium and high MOIs, which correlated with a decrease in cell viability as the infection progressed in our study (Figure 2). This phenomenon was similar to a previous study that demonstrated a dramatic decrease in the cell viability of neuroblastoma HTB-11 cells on day 3 after infection with the JEV Nakayama-HIH strain. In addition, almost 100% of JEV-infected cells died 5 days after infection [6].

In terms of virus replication, the SH-SY5Y cells were permissive cells and allowed JEV to produce new virus progeny. The amount of new JEV progeny at an MOI of 1 continuously increased to a maximum level at 48 hpi and reached a plateau at 72 hpi, which corresponded to the accumulation of apoptotic cells. The present kinetics of the replication pattern of JEV in SH-SY5Y cells was consistent with previous reports in other neuroblastoma cell lines, such as human neuroblastoma HTB-11 cells [6], TE671 human medulloblastoma cells [11], mouse neuroblastoma N18 cells [25], and mouse neuroblastoma N2a cells [26]. Yang et al. [6] showed that JEV (Nakayama-NIH strain) at an MOI of 1 replicated effectively in HTB-11 cells and induced extensive cell apoptosis within 72 hpi. Moreover, it has been proven that the progression of JEV replication is associated with the induction of apoptosis in N2a cells [26].

Apoptotic cell death by JEV infection has been described both in vitro and in vivo. Japanese encephalitis virus-induced apoptosis can be triggered by multiple signaling pathways and regulated by multiple, complex extrinsic and intrinsic ligands [27]. Previous studies have reported that JEV replication causes the accumulation of misfolded proteins in the endoplasmic reticulum (ER) lumen and triggers ER stress, subsequently activating apoptosis via the IRE1/JNK pathway in BHK-21 cells [28]. Japanese encephalitis virus infection increases intracellular ROS and activates ERK/p38 MAPK signal transduction to induce apoptosis [29]. Japanese encephalitis virus infection induced N2a cell apoptosis by downregulating the STAT3-FoxO-Bcl-6/p21 pathway [12]. Japanese encephalitis virus infection induced oxidative stress, which elicited the release of Cyt *c* from mitochondria and resulted in the induction of apoptosis, which is associated with the roles of Bcl-2 family proteins [25,30,31].

Our present study reports for the first time the kinetics of the expression of two Bcl-2 family proteins during JEV infection. The proapoptotic Bcl-2-associated X protein—or Bax—is the active effector of caspase-3 that is able to induce apoptosis. Here, we observed two distinct expression profiles of Bax dependent on the dose of JEV virus and the stage of infection. At low MOI of JEV, the expression levels of full-length p21 Bax were high, whereas very little p18 Bax was detected by Western blot and only in the late stage of infection (Figure 4). The cleavage fragment of p18 Bax was generated with the downregulation of full-length p21 Bax at the late stages of 48 and 72 hpi following high MOI of JEV. The slight induction in p21 Bax expression in the JEV-infected SH-SY5Y cells was observed only at 24 hpi at a low MOI but not at a high MOI. A similar result of p21 Bax expression was observed in human brain endothelial cells (THBMECs) during JEV (Nakayama strain) infection [13]. The reduction in full-length p21 Bax expression may be due to the proteolytic processing that generates the cleavage fragment of p18 Bax. Interestingly, we found that p18 Bax formation appeared to correlate with the JEV induction of apoptotic cell death, as observed by the increase in the proportion of apoptotic cells via annexin V analysis and the increase in cell death via cell viability assay, together with the release of Cyt *c* and the activation of caspase-3/7 activity, especially during the late stage of a robust viral infection.

The cleavage of Bax at the N-terminal region by calpain, a calcium-dependent cysteine protease [16], has been implicated in several types of stimuli that induce apoptosis in a variety of different cell types, such as drugs or toxic agents [19,32,33,34,35], interferon alpha [36], pollutants [37], and viral infections [38,39]. Previous reports indicated that the cleaved form p18 Bax was more apoptogenic than the full-length p21 Bax for drug-induced cell death in several cell types [16,20,21,40]. The co-transfection experiments have shown that the induction of apoptotic cell death of the p18 Bax was substantially resistant to Bcl-x_L_- or Bcl-2-mediated rescue compared with full-length of Bax, in spite of the complex formation among these two molecules [20,32,40]. The N-terminal truncated Bax subunit directly mediates Cyt *c* release into the cytoplasm and initiates the downstream signaling cascade of mitochondrial-mediated apoptosis [16,32]. In viral infection, our present study showed, for the first time, the difference in p18 Bax generation in JEV Beijing-1 infection that was dependent on the viral load and the period of infection. The p18 Bax expression was also found in cells infected with other viruses, such as vesicular stomatitis virus [38], Sindbis virus, and Semliki Forest virus [39]. Moreover, we also showed that JEV infection mainly induced the accumulation of mitochondrial p18 Bax which caused the release of Cyt *c* into the cytosol and then subsequently induced SH-SY5Y cells to undergo apoptosis at the late stage of infection, corresponding to a previous report of stress agents-induced apoptosis in dopaminergic neuronal cell line [19]. The increase in p18 Bax after virus infection led to the amplification of the apoptotic process in the infected cells [38]. Therefore, our results suggest another possible mechanism of JEV-induced cell death via the induction of the proteolysis of endogenous p21 Bax to generate p18 Bax during apoptosis. The p21 Bax itself can initiate the mitochondrial apoptotic pathway while p18 Bax potently accelerates the apoptosis by disrupting mitochondrial integrity. The formation of p18 Bax increases the intrinsic cytotoxic properties of this proapoptotic p18 Bax molecule that could enhance its function in the mitochondria to promote cell death.

Several reports have demonstrated that JEV infection induces not only host cell apoptosis but also cell survival in order to establish viral persistence [17,41,42]. Anti-apoptotic protein Bcl-2 has been shown to play a role in the protection from apoptosis during flavivirus infection [41,42]. The present study shows that JEV slowly stimulated the expression of Bcl-2 protein during the early period of infection. The expression level of Bcl-2 was slightly increased when cells were infected with a high MOI of JEV when compared with uninfected cells. The kinetic viability and apoptosis analysis clearly showed an increase in cells undergoing apoptosis and cell death, together with the induction of caspase activity in the cells infected with 1 MOI of JEV. The results suggested that Bcl-2 did not effectively suppress JEV-induced cell death during the late period of infection. As observed in a previous study, high expression of Bcl-2 was detected in JEV-infected THBMECs cells [13]. The enforced expression of Bcl-2 protein could not inhibit JEV-induced apoptosis and failed to block virus replication in N18 cells and BHK-21 cells [43]. In addition, the overexpression of Bcl-2 in JEV-infected cells was demonstrated to be related to the inhibition of apoptosis at the early stage of infection to facilitate persistent infection [41,42]. Therefore, it is possible that the expression of Bcl-2 may play a role in protecting cells and delays JEV-induced apoptosis, especially in the early stage of infection but not in the later stage of infection.

In this study, we clearly demonstrated that JEV induced accumulation of p18 Bax leading to the neuronal apoptosis in neuroblastoma SH-SY5Y cells in vitro. Further in vivo studies using animal model would provide more evidence that the elevation of p18 Bax levels by JEV infection plays a crucial role in neuronal cell apoptosis with changes in brain tissue, such as those identified in our in vitro study. The histopathological changes of brain tissue observed by immunohistochemistry and the TUNEL assay, as well as changes of p18 Bax levels in total brain tissue protein or the mitochondrial and cytosolic fraction of brain tissue analyzed by Western blot analysis, might be important to provide novel insights into JEV-caused neuronal apoptosis and encephalitis. In summary, we have revealed the kinetics of viral replication and the neuronal cell apoptosis pattern of JEV infection in human neuroblastoma SH-SY5Y cells. Japanese encephalitis virus-induced neuronal cell death is a major mechanism involved in viral pathogenesis. Our study demonstrated that upon JEV infection, the induction of apoptotic signal molecules slowly progressed to induce the cell death process at the early stage of infection, which may be due to the protective role of anti-apoptotic Bcl-2 proteins. The cleavage form of the proapoptotic Bax protein, p18 Bax, was remarkably present during the late stage of JEV infection, which enhanced its cell death function by the mitochondria-mediated apoptosis pathway. Thus, the increase in p18 Bax by JEV infection influenced neuronal cell death. Our findings provide important information to facilitate further studies on the development of novel therapeutic treatments for JEV infection. The discovery of new lead compounds that could block the proteolysis of p21 Bax for preventing the generation of p18 Bax or directly inhibiting the potent proapoptotic activity of the cleaved p18 Bax could be used to reduce neuronal apoptosis by JEV infection. The combination of antiviral replication agents and neuroprotective agents could provide an alternative treatment strategy for alleviating neurological sequelae from JEV-induced encephalitis.

## 4. Materials and Methods

### 4.1. Cell Culture

The SH-SY5Y cells (human neuroblastoma, ATCC No. CRL-2266) were used as representative of the neuronal target cell of JEV. The SH-SY5Y cells were cultured in Dulbecco’s modified Eagle’s medium (DMEM) supplemented with 10% heat-inactivated fetal bovine serum (FBS). Cells were maintained in a humidified incubator at 37 °C in a humidified atmosphere containing 5% CO_2_. The SH-SY5Y cultures were maintained and sub-cultured to obtain the number of cells for the next experiment.

### 4.2. JEV Propagation in Vero Cells

The Beijing-1 strain of JEV was kindly provided by Sutee Yoksan at the Center for Vaccine Development, Institute of Molecular Biosciences, Mahidol University. The virus was identified and the JEV strain was confirmed by performing RT-PCR and sequence analysis (data not shown). Then, JEV was propagated in Vero cells (African green monkey kidney cells, ATCC No. CCL-81). Vero cell monolayers were inoculated with JEV at the appropriate MOI and incubated at 37 °C and 5% CO_2_ for 1–2 h for virus adsorption. The inoculum was removed, and the cells were washed with 2% FBS-supplemented Eagle’s minimum essential medium (EMEM). The infected cell cultures were maintained in 2% FBS EMEM until the infected monolayers showed 3 to 4+ degrees of cytopathic effect (CPE). Subsequently, the culture supernatant was harvested by centrifugation at 2000 rpm for 20 min at 4 °C and aliquoted to generate a virus stock. Virus stock was stored at −80 °C, and virus titer was determined by plaque assay.

### 4.3. Virus Titration Assay

The JEV titer was determined by plaque assay. Briefly, Vero cells were seeded in a 12 well culture plate. After 24 h, Vero cell monolayers were infected with serial 10 fold dilutions of virus in triplicate. The inoculated cells were incubated at 37 °C and 5% CO_2_ for 1 h with intermittent shaking for virus adsorption. The monolayer was overlaid with 1.5% carboxymethylcellulose in 2% FBS EMEM and incubated at 37 °C and 5% CO_2_ for 6 days. The cell monolayer was stained with crystal violet after fixing the cells with 10% formaldehyde. The number of plaques was counted and calculated as plaque forming units (pfu/mL). The average from triplicate wells is presented.

### 4.4. JEV Infection in Neuronal SH-SY5Y Cells

The SH-SY5Y cells were seeded in 24 well plates for 24 h. Then, the SH-SY5Y cells were infected with JEV at an MOI of 0.01, 0.1, and 1 for 1 h. After virus adsorption, infected cells were washed with 1× DMEM and maintained in 2% FBS in DMEM for 24, 48, and 72 h. The JEV-infected cells were collected to perform indirect immunofluorescence assay (IFA) using anti-flavivirus enveloped protein (4G2) antibody (MilliporeSigma, Burlington, MA, USA) followed by incubation with a secondary antibody, fluorescein isothiocyanate (FITC)-conjugated goat anti-mouse IgG in Evan’s blue solution (MilliporeSigma). The cells were imaged using a Nikon ECLIPSE 80i fluorescence microscope (Nikon, Tokyo, Japan). The viral progeny in the culture supernatant were harvested and titers were determined by plaque assay.

### 4.5. Cell Viability Assay

Cell viability was determined using the CellTiter 96^®^ AQueous One Solution Cell Proliferation assay (Promega, Madison, WI, USA). The SH-SY5Y cells were seeded in 96 well plates for 24 h and then infected with JEV as described above. At 24, 48, and 72 h post infection (hpi), the assay reagent was added to the infected cells and incubated at 37 °C and 5% CO_2_ for 3 h. The optical density (OD) at a wavelength of 490 nm was measured by a Synergy HTX Multi-Mode Reader (BioTek, Winooski, VT, USA). The percentage of cell viability was calculated relative to control cells designated as 100% viable cells using the following formula:

(1) $$\left(\frac{A_{\mathrm{JEV}}-A_{B}}{A_{C}-A_{B}}\right)\times 100=\%\;\mathrm{cell}\;\mathrm{viability}$$

where A_JEV_ = absorbance value of JEV-infected cells, A_B_ = absorbance value of blank (medium alone), A_C_ = absorbance value of control cells.

### 4.6. Western Blot Analysis

The SH-SY5Y cells were collected at 24, 48, and 72 hpi. The infected cells were lysed in RIPA buffer containing a protease inhibitor cocktail, homogenized, and centrifuged at 10,000× *g* for 15 min at 4 °C. After centrifugation, the supernatant was collected, and the total protein concentration was measured using a Bradford assay (Bio-Rad Laboratories, Hercules, CA, USA). Equal amounts of protein extracts were separated on polyacrylamide gels and transferred to polyvinylidene difluoride (PVDF) membranes for immunoblotting. The membranes were blocked for 1 h at room temperature in 5% non-fat dry milk in tris-buffered saline with Tween 20 (TBST) and then incubated overnight with either a rabbit monoclonal anti-humam Bax antibody (1:1000, Cell Signaling Technology, Inc., Danvers, MA, USA), mouse monoclonal anti-human Bcl-2 antibody (1:1000, Cell Signaling Technology, Inc.), or mouse monoclonal anti-human actin antibody (1:10,000, MilliporeSigma). After washing with TBST, the membranes were incubated with horseradish peroxidase (HRP)-conjugated anti-rabbit IgG (Cell Signaling Technology, Inc.) or anti-mouse IgG (Cell Signaling Technology, Inc.) antibodies for 1 h at room temperature. Finally, immunoblots were visualized using chemiluminescent HRP substrates (MilliporeSigma). The band intensities were quantified by densitometry using ImageJ software (NIH, Bethesda, MD, USA).

### 4.7. Apoptotic Cell Death Analysis

Apoptosis in JEV-infected and uninfected cells at 24, 48, and 72 hpi was assayed using the Muse^®^ Annexin-V and Dead Cell Kit from MilliporeSigma (Cat# MCH100105). Briefly, cells were stained with annexin V, an apoptotic cell dye, and 7-AAD, a cell death dye, and were analyzed using a Muse^®^ Cell Analyzer (MilliporeSigma). Apoptosis and cell death rate were presented as percentages of total cells.

### 4.8. Cytochrom c Releasing Assay

In order to ensure that JEV induced neuronal apoptosis via a mitochondria-dependent pathway, the translocation of Cyt *c* from mitochondria into cytosol was determined. The cytosolic and mitochondrial fractionations of JEV-infected cells were prepared using the Mitochondria/Cytosol Fractionation Kit (Abcam, Cambridge, UK) according to the instructions of the manufacturer. Briefly, approximately 6 × 10^6^ SH-SY5Y cells were infected with JEV at an MOI of 1 as described in 4.4 then the JEV-infected cells were harvested at 24, 48, and 72 hpi. The cells were gently pelleted by low-speed centrifugation (600× *g*), washed once in ice-cold PBS, pelleted again, and re-suspend cells with 1× Cytosol Extraction Buffer Mix containing protease inhibitors and DTT and incubated on ice for 10 min. Cells were subjected for homogenization with a 27 G gauge syringe. The efficiency of homogenization was checked by microscopy when 70–80% of the cells did not have the shiny ring. Then, the homogenates were transferred to a 1.5 mL microcentrifuge tube and centrifuged (700× *g*) for 10 min at 4 °C. The supernatants were transferred to a fresh microcentrifuge tube and centrifuged at 10,000× *g* for 30 min at 4 °C. In this step, supernatants were collected as cytosolic fractions. The pellets were re-suspended with the Mitochondrial Extraction Buffer Mix containing protease inhibitors and DTT and mixed by vortex for 10 s, then this solution was designated as mitochondrial fractions. The presence of Cyt *c* was determined in both the mitochondrial and cytosol extractions by Western blotting, using a rabbit monoclonal anti-human Cyt *c* antibody (1:1000, Cell Signaling Technology, Inc.). The purity of mitochondrial and cytosolic fractions was confirmed by using anti-cytochrome oxidase IV (Cox IV) (1:2500, Cell Signaling Technology, Inc.) and anti-Actin (1:10,000, MilliporeSigma), respectively.

### 4.9. Caspase Activity Assay

The activity of caspase was determined in JEV-infected and uninfected cells at 24, 48, and 72 hpi by Muse™ Caspase-3/7 Assay (Cat# MCH100108, MilliporeSigma) according to the manufacturer’s protocol. Briefly, cells were incubated with fluorogenic Muse™ Caspase-3/7 reagent for detecting caspase-3/7 activity at 37 °C for 30 min and were analyzed using a Muse^®^ Cell Analyzer (MilliporeSigma).

### 4.10. Statistical Analysis

Data are presented as the mean ± SD from three to five independent experiments. The significance of the differences among groups was evaluated by using one- or two-way analysis of variance (ANOVA) followed by a multiple comparison test as appropriate. Graphs were plotted and analyzed using GraphPad Prism (v6.0, GraphPad, San Diego, CA, USA). A *p*-value less than 0.05 was considered statistically significant.
